# Supplementary material for: Dietary Regulation of Lipid Metabolism in Gestational Diabetes Mellitus: Implications for Fetal Macrosomia
Source: Int J Mol Sci. 2024 Oct 19;25(20):11248. doi: 10.3390/ijms252011248 (PMC11508696; doi:10.3390/ijms252011248)
Supplement: Supplementary file 1 [file ijms-25-11248-s001.zip › ijms-3241861-supplementary.pdf]

International Journal of *Molecular Sciences*

MDPI

Article

Dietary Regulation of Lipid Metabolism in Gestational Diabetes Mellitus: Implications for Fetal Macrosomia

Natalia Frankevich[1,*], Vitaly Chagovets[1], Alisa Tokareva[1], Natalia Starodubtseva[1,2], Elizaveta Limonova[1], Gennady Sukhikh[1,3] and Vladimir Frankevich[1,4]

[1] National Medical Research Center for Obstetrics Gynecology and Perinatology Named after Academician V.I. Kulakov of the Ministry of Healthcare of Russian Federation, 117997 Moscow, Russia
[2] Moscow Center for Advanced Studies, 123592 Moscow, Russia
[3] Department of Obstetrics, Gynecology, Perinatology and Reproductology, Institute of Professional Education, Federal State Autonomous Educational Institution of Higher Education I.M. Sechenov First Moscow State Medical University of the Ministry of Health of the Russian Federation (Sechenov University), 119991 Moscow, Russia
[4] Laboratory of Translational Medicine, Siberian State Medical University, 634050 Tomsk, Russia
Correspondence: n_frankevich@oparina4.ru

Table S1. Summary of clinical characteristics of the group. * Statistically significant differences and statistically significant p-values. Clinical parameters and p-values with statistical significance are highlighted in bold.

Parameter	Group		Value	*P-value*	Value	*P-value*
**Age, years**	GDM	GDM, diet	32 (29;35)	0.56	31 (27; 33)	0.08
		GDM, no-diet			37 (31; 39)	
	Control		31 (28; 34)			
**Weight before pregnancy, kg**	GDM	GDM, diet	64 (55;77)	0.13	58 (54; 65)	**0.004***
		GDM, no-diet			81 (66; 86)	
	Control		60 (55; 25)			
**Height, sm**	GDM	GDM, diet	168 (164;170)	0.91	168 (164; 170)	0.20
		GDM, no-diet			170 (164; 179)	
	Control		168 (164; 171)			
**BMI before pregnancy, kg/m²**	GDM	GDM, diet	22.6 (20.1;26.5)	**0.03***	20.1 (19.7; 23.5)	**0.003***
		GDM, no-diet			26.4 (24.5; 27.6)	
	Control		21.2 (19.5; 22.9)			
**Weight gain at birth, kg**	GDM	GDM, diet	14 (11;17)	**0.009***	11 (9; 13)	**0.006***
		GDM, no-diet			13 (10; 16)	
	Control		11(9;15)			
	GDM	GDM, diet	3.5 (3.2;3.8)	0.86	3.5 (3.2;3.7)	0.41
		GDM, no-diet	3.5 (3.2;3.7)		3.5 (3.4;3.9)	

**Patient's newborn weight, kg**	Control					
**Husband's newborn weight, kg**	GDM	GDM, diet	3.6 (3.4;3.9)	0.78	3.6 (3.5; 3.9)	0.89
		GDM, no-diet			3.7 (3.4; 3.8)	
	Control		3.6(3.4;3.9)			
**First-birth patients, n (%)**	GDM	GDM, diet	13 (43%)	0.67	11 (55%)	0.15
		GDM, no-diet			2 (20%)	
	Control		30 (38%)			
**Fetal weight according to ultrasound (32 weeks), g**	GDM	GDM, diet	1916(1738;	0.24	1899(1717; 2110)	**0.04***
		GDM, no-diet			2132(1865;229)	
	Control		1830(1568;2005)			
**Delivery date, weeks**	GDM	GDM, diet	39.0 (38.4;39.6)	0.17	39.3(38.4;39.7)	0.08
		GDM, no-diet			38.3(38.0;39.4)	
	Control		39.5(38.4;40.2)			
**Operative delivery, number of patients (%)**	GDM	GDM, diet	18 (60%)	**0.02***	11 (55%)	0.69
		GDM, no-diet			7 (70%)	
	Control		27 (34%)			
**Planned operative delivery, number of patients (%)**	GDM	GDM, diet	13 (43%)	**0.01***	ПКС – 7 (35%)	0.42
		GDM, no-diet			ПКС – 6 (60%)	
	Control		14 (18%)			
**Induction of labor, number of patients (%)**	GDM	GDM, diet	6(20%)	1	3(15%)	0.62
		GDM, no-diet			3(30%)	
	Control		16 (20%)			
**Time of discharge of the woman in labor, days**	GDM	GDM, diet	5(3;5)	**0.02***	5(3;5)	0.92
		GDM, no-diet			5 (3;5)	
	Control		4(3;5)			

**Newborn weight, grams**	GDM	GDM, diet	3398(3202;3873)	0.42	3313(3197;376)	0.14
	Control	GDM, no-diet	3495(3207;4032)		3810(3317;4122)	
**Apgar score—1 minute, number of patients (%)**	GDM	GDM, diet	8[8;8]	0.53	8[8;8]	0.33
	Control	GDM, no-diet	8[8;8]		8[8;8]	
**Apgar score—5 minutes, number of patients (%)**	GDM	GDM, diet	9[9;9]	0.74	9[9;9]	0.71
	Control	GDM, no-diet	9[9;9]		9[9;9]	
**Complications of the early neonatal period, number of patients (%)**	GDM	GDM, diet	15(50%)	0.32	12 (60%)	0.24
	Control	GDM, no-diet	30 (38%)		3 (30%)	
**Newborn discharge dates**	GDM	GDM, diet	4[3;5]	0.15	4[3;5]	0.82
	Control	GDM, no-diet	3[3;4]		4[3;5]	

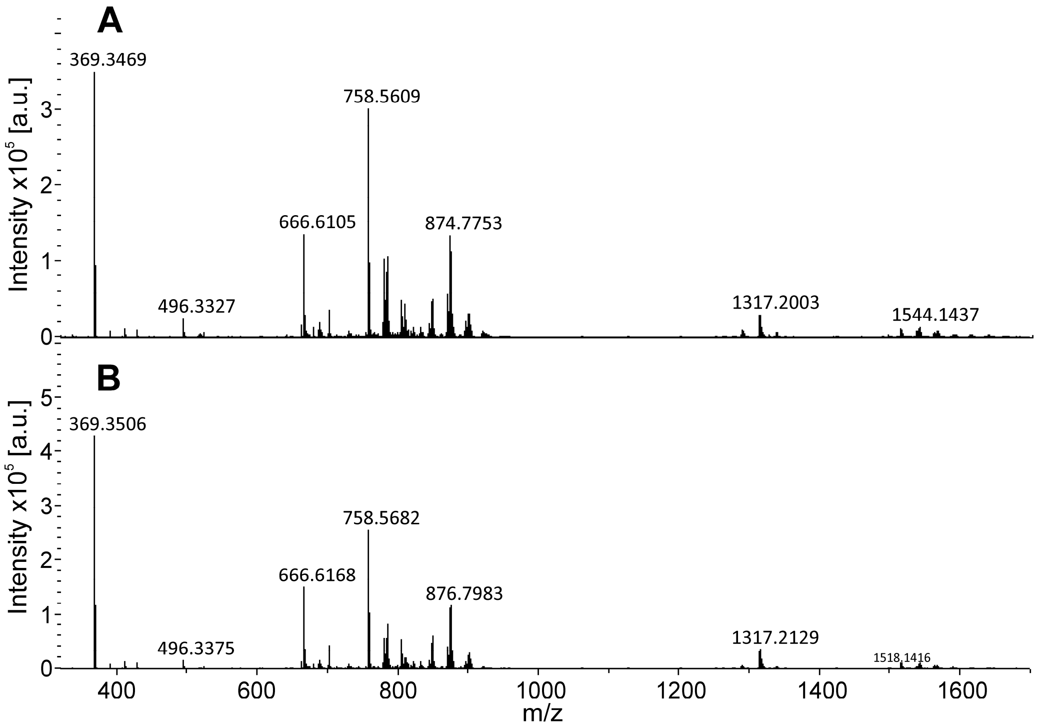

Figure S1. Positive ion mass spectra of plasma lipid extracts. A) Plasma from patients adhering to a diet; B) plasma from patients not adhering to a diet.

Table S2. Lipid levels that were most significant for classifying patients who adhered to the diet and those who did not. The data are provided for the model based on the analysis of samples obtained at 11–13 weeks.

Lipid	Adhered to the diet	Did not adhere to the diet	p-value
PC 34:2	0.0362 (0.035; 0.0406)	0.0435 (0.0403; 0.0475)	0.0025
PC 36:2	0.0142 (0.0126; 0.0163)	0.0168 (0.0153; 0.0179)	0.051
PC 38:3	0.0032 (0.003; 0.0036)	0.0027 (0.0022; 0.0029)	9.00E-04
PC 36:3	0.011 (0.0103; 0.0113)	0.0106 (0.0095; 0.0118)	0.5241
PC 34:1	0.0144 (0.0138; 0.0163)	0.015 (0.014; 0.0159)	0.9812
PC 36:4	0.0166 (0.0144; 0.0193)	0.0163 (0.0149; 0.018)	0.7595
PC 32:1	9e-04 (7e-04; 0.001)	7e-04 (6e-04; 7e-04)	0.0717
PC 38:4	0.0075 (0.0071; 0.0092)	0.0072 (0.0063; 0.0083)	0.3816
PC 36:5	0.0022 (0.0021; 0.0035)	0.0026 (0.0023; 0.0036)	0.6888
PC 38:6	0.0069 (0.0065; 0.0081)	0.0081 (0.0074; 0.0092)	0.2259
PC O-36:3	2e-04 (0; 4e-04)	5e-04 (5e-04; 6e-04)	0.0057
SM 34:1	0.0064 (0.006; 0.0068)	0.0068 (0.0062; 0.0079)	0.2079
SM 42:2	0.0032 (0.0029; 0.0038)	0.0038 (0.0035; 0.004)	0.051
SM 46:0	0.0042 (0.0036; 0.005)	0.0035 (0.0023; 0.0053)	0.6206
SM 42:1	0.0017 (0.0016; 0.0019)	0.0019 (0.0017; 0.002)	0.3562

Table S3. Lipid levels that were most significant for classifying patients who adhered to the diet and those who did not. The data are provided for the model based on the analysis of samples obtained at 24–26 weeks.

**Lipid**	**Adhered to the diet**	**Did not adhere to the diet**	**p-value**
LPC 16:0	0.0039 (0.0038; 0.0043)	0.0032 (0.0026; 0.0037)	0.0072
PC 34:2	0.0434 (0.0405; 0.0453)	0.0457 (0.0427; 0.052)	0.1599
PC 36:2	0.0138 (0.013; 0.0145)	0.015 (0.0136; 0.0167)	0.08
PC O-36:3	2e-04 (0; 4e-04)	5e-04 (4e-04; 5e-04)	0.0523
SM 46:0	0.0059 (0.0047; 0.0066)	0.0061 (0.0038; 0.008)	0.9437
SM 48:1	0.0022 (0.0017; 0.0024)	0.0024 (0.0017; 0.0028)	0.5554
SM 48:2	8e-04 (7e-04; 0.001)	0.0011 (6e-04; 0.0012)	0.3562
SM 34:1	0.0059 (0.0057; 0.0067)	0.0069 (0.0062; 0.0072)	0.1092
SM 47:3	6e-04 (5e-04; 8e-04)	5e-04 (4e-04; 6e-04)	0.0987
SM 48:0	0.0034 (0.0029; 0.004)	0.0038 (0.0025; 0.0045)	0.6544

Table S4. Lipid levels that were most significant for classifying patients who adhered to the diet and those who did not. The data are provided for the model based on the analysis of samples obtained at 30–32 weeks of pregnancy.

**Lipid**	**Adhered to the diet**	**Did not adhere to the diet**	**p-value**
LPC 16:0	0.002 (0.0018; 0.0023)	0.0016 (0.0015; 0.002)	0.1061
PC 34:2	0.0424 (0.0405; 0.0448)	0.0498 (0.0458; 0.0527)	0.0019
PC 36:2	0.0134 (0.012; 0.014)	0.0141 (0.0133; 0.0155)	0.0849
PC 36:4	0.0146 (0.0124; 0.0155)	0.0124 (0.0113; 0.0146)	0.2746
PC 38:3	0.002 (0.0019; 0.0025)	0.0018 (0.0017; 0.0021)	0.0757
PC 38:4	0.005 (0.0045; 0.0057)	0.0043 (0.004; 0.0054)	0.0849
PC 32:1	8e-04 (7e-04; 0.001)	8e-04 (6e-04; 8e-04)	0.2525
PC O-36:3	0 (0; 3e-04)	4e-04 (4e-04; 5e-04)	0.019
PC O-34:1	3e-04 (2e-04; 3e-04)	3e-04 (3e-04; 4e-04)	0.0062
SM 46:0	0.0048 (0.0044; 0.0061)	0.0052 (0.0049; 0.0093)	0.3751
SM 48:0	0.0034 (0.0027; 0.0035)	0.0037 (0.0032; 0.0044)	0.0951
SM 48:1	0.0018 (0.0016; 0.0022)	0.002 (0.0018; 0.0028)	0.3751
SM 34:1	0.0062 (0.0059; 0.0066)	0.0064 (0.0061; 0.0071)	0.2746
TG 52:0	7e-04 (6e-04; 7e-04)	5e-04 (4e-04; 6e-04)	0.0268
